# Identification of YbeY-Protein Interactions Involved in 16S rRNA Maturation and Stress Regulation in *Escherichia coli*

**DOI:** 10.1128/mBio.01785-16

**Published:** 2016-11-08

**Authors:** Maarten Vercruysse, Caroline Köhrer, Yang Shen, Sandra Proulx, Anubrata Ghosal, Bryan W. Davies, Uttam L. RajBhandary, Graham C. Walker

**Affiliations:** a Department of Biology, Massachusetts Institute of Technology, Cambridge, Massachusetts, USA; b Department of Electrical and Computer Engineering, TEES-AgriLife Center for Bioinformatics and Genomic Systems Engineering, Texas A&M University, College Station, Texas, USA; c Department of Molecular Biosciences, University of Texas at Austin, Austin, Texas, USA

## Abstract

YbeY is part of a core set of RNases in *Escherichia coli* and other bacteria. This highly conserved endoribonuclease has been implicated in several important processes such as 16S rRNA 3′ end maturation, 70S ribosome quality control, and regulation of mRNAs and small noncoding RNAs, thereby affecting cellular viability, stress tolerance, and pathogenic and symbiotic behavior of bacteria. Thus, YbeY likely interacts with numerous protein or RNA partners that are involved in various aspects of cellular physiology. Using a bacterial two-hybrid system, we identified several proteins that interact with YbeY, including ribosomal protein S11, the ribosome-associated GTPases Era and Der, YbeZ, and SpoT. In particular, the interaction of YbeY with S11 and Era provides insight into YbeY’s involvement in the 16S rRNA maturation process. The three-way association between YbeY, S11, and Era suggests that YbeY is recruited to the ribosome where it could cleave the 17S rRNA precursor endonucleolytically at or near the 3′ end maturation site. Analysis of YbeY missense mutants shows that a highly conserved beta-sheet in YbeY—and not amino acids known to be important for YbeY’s RNase activity—functions as the interface between YbeY and S11. This protein-interacting interface of YbeY is needed for correct rRNA maturation and stress regulation, as missense mutants show significant phenotypic defects. Additionally, structure-based *in silico* prediction of putative interactions between YbeY and the Era-30S complex through protein docking agrees well with the *in vivo* results.

## IMPORTANCE

Ribosomes are ribonucleoprotein complexes responsible for a key cellular function, protein synthesis. Their assembly is a highly coordinated process of RNA cleavage, RNA posttranscriptional modification, RNA conformational changes, and protein-binding events. Many open questions remain after almost 5 decades of study, including which RNase is responsible for final processing of the 16S rRNA 3′ end. The highly conserved RNase YbeY, belonging to a core set of RNases essential in many bacteria, was previously shown to participate in 16S rRNA processing and ribosome quality control. However, detailed mechanistic insight into YbeY’s ribosome-associated function has remained elusive. This work provides the first evidence that YbeY is recruited to the ribosome through interaction with proteins involved in ribosome biogenesis (i.e., ribosomal protein S11, Era). In addition, we identified key residues of YbeY involved in the interaction with S11 and propose a possible binding mode of YbeY to the ribosome using *in silico* docking.

## INTRODUCTION

The highly conserved RNase YbeY plays a critical role in multiple cellular processes. To date, studies have shown that YbeY is crucial in 16S rRNA maturation, 70S ribosomal assembly, late-stage 70S ribosome quality control, and stress regulation in *Escherichia coli*, *Vibrio cholerae*, *Sinorhizobium meliloti* and chloroplasts of *Arabidopsis thaliana* (1–5). In addition to a general role in mRNA regulation (6), YbeY is also implicated in the bacterial small RNA (sRNA) regulatory network, acting alongside key proteins such as Hfq, RNase E, or PNPase (2, 7–9). It is, therefore, not surprising that YbeY has an important role in host-microbe interactions. We have shown earlier that the *S. meliloti* homolog of YbeY is essential to establish the chronic intracellular infection necessary for symbiosis of *S. meliloti* with alfalfa (5). In *V. cholerae*, YbeY is involved in pathogenesis, as its depletion leads to reduced colonization of mouse intestines and decreased cholera toxin production (2). Moreover, inactivation of YbeY was recently shown to impair many virulence-related features of *Yersinia enterocolitica* (10). Yet another striking example for YbeY’s central role in bacterial RNA metabolism is its association with apoptosis-like death (ALD) in *E. coli* in response to severe DNA damage, which—among other specific characteristics—also shows YbeY-dependent rRNA degradation (11).

Despite the growing number of broad phenotypical studies, detailed mechanistic insight into YbeY’s functions, particularly the functions that are associated with rRNA maturation and the ribosome, has remained limited. Ribosome biogenesis is a highly coordinated process involving ribosomal proteins, numerous RNases, RNA modification enzymes, and assembly factors that mediate rRNA maturation and assembly into ribosomal subunits and fully functional ribosomes (12–14). Processing of the rRNA precursor into mature sequences involves the precise action of a series of RNases, including YbeY (1–3, 15). Many open questions remain after almost 5 decades of study, including which RNase is responsible for maturation of the 3′ terminus of 16S rRNA. So far, YbeY has been the only endonuclease implicated in the maturation of the all-critical 16S rRNA 3′ terminus (1–3).

Considering YbeY’s multifaceted role within the cell and the fact that it belongs to a core set of RNases essential in many bacteria, it is likely that YbeY interacts with many of the key players in RNA metabolism and ribosome biogenesis. The identification of protein-protein interactions and functional associations is critical to understand specific cellular processes as well as their interconnectedness. During the last decade, a small number of system-wide studies in *E. coli* attempted to provide insight into the overall architecture of pathways and functional modules mediating cellular processes. The proteome-wide studies by Butland et al. (16) in 2005 and Arifuzzaman et al. (17) in 2006 used a combined approach of affinity purification of proteins and mass spectrometry (AP-MS) to uncover the interaction network of protein complexes. To elucidate the biological role of functionally unannotated proteins (orphans), in 2009 Hu et al. (18) combined proteomics and genomic context (GC) analysis tools to map orphans to specific biological processes. Examining genetic interactions (GI), or epistasis, can also provide functional relationship and pathway redundancies via phenotypic analysis of double mutants (19, 20). The loss of two genes may show an aggravating GI if they functionally compensate each other. In contrast, yeast two-hybrid analysis Y2H can, unlike AP-MS, GC, and GI, identify direct binary protein-protein interactions and provide information about the internal topology of protein complexes independent of the conditions in *E. coli*. In 2014, using Y2H, Rajagopala et al. (21) estimated the total *E. coli* interactome to be on the order of ~10,000 protein-protein interactions.

Bacterial two-hybrid analysis B2H is even more sensitive than Y2H in detecting bacterial multiprotein complexes, since B2H is carried out in the native bacterial environment that also contains additional associated proteins or other cofactors (22). B2H or BACTH (bacterial adenylate cyclase-based two-hybrid system) was originally developed by Karimova et al. (23) in 1998 and has been used in numerous studies to obtain detailed mechanistic data. For example, Battesti et al. (24, 25) used BACTH to show how the acyl carrier protein interacts with the stress regulator SpoT to trigger the stringent response depending on the status of the cellular fatty acid metabolism in beta- and gammaproteobacteria. B2H studies also identified the components of the RNA degradosome-like complex in *Staphylococcus aureus* and *Bacillus subtilis*, showing the association of glycolytic enzymes with RNA processing and degrading enzymes (26, 27).

In this paper, we have used B2H to identify several key protein-protein interactions that provide further insight into YbeY’s function. In particular, we showed that YbeY interacts with the ribosomal protein S11, the ribosome-associated GTPase Era and GTPase Der, YbeZ, and the stringent response regulator SpoT. Mutagenesis analyses demonstrated that YbeY’s protein-protein interaction interface with S11 is distinct from its catalytic domain and is required for correct processing of the 16S rRNA 3′ terminus. Moreover, disruption of the protein-protein interaction interface also resulted in a pleiotropic phenotype of *E. coli* under stress conditions. Structure-based *in silico* prediction of the interaction between YbeY and the ribosome further supported YbeY’s role in ribosomal maturation and quality control.

## RESULTS

### Identification of potential interaction partners of the bacterial RNase YbeY.

To gain further mechanistic insight into YbeY’s complex function, we used B2H to identify proteins that interact with YbeY. We focused on a limited number of candidate interaction partners, chosen on the basis of possible functional association correlated with the role of YbeY in rRNA maturation, ribosome biogenesis, and stress regulation (1–3) (Table 1).

**TABLE 1:** YbeY candidate interactors

Category and protein^a^	General description^a^	B2H interaction with YbeY^b^
Ribosome-associated GTPases		
Era	GTPase involved in ribosome biogenesis	+
Der	GTPase involved in ribosome biogenesis	+
ObgE	GTPase involved in ribosome biogenesis	−
MazG	Nucleoside triphosphate pyrophosphohydrolase	−
Ribosomal assembly cofactors		
KsgA	rRNA small-subunit methyltransferase A	−
RsgA	Putative ribosome biogenesis GTPase RsgA	−
RbfA	Ribosome-binding factor A	−
RimM	Ribosome maturation factor RimM	−
Ribosomal proteins		
S1	Ribosomal protein S1	−
S2	Ribosomal protein S2	−
S7	Ribosomal protein S7	−
S11	Ribosomal protein S11	+
S18	Ribosomal protein S18	−
Stringent response		
SpoT	Bifunctional (p)ppGpp synthase/hydrolase	+
RelA	Bifunctional (p)ppGpp synthase/hydrolase	−
DksA	RNA polymerase-binding transcription factor	−
BipA	GTP-binding protein	−
“Other” candidate interactors		
RNase E	RNase E	−
RNase R	RNase R	−
PNPase	Polynucleotide phosphorylase	−
RhlB	ATP-dependent RNA helicase	−
IF2	Initiation factor 2	−
IF3	Initiation factor 3	−
YbeZ	PhoH-like protein	+
MazE	Antitoxin component of MazE-MazF module	−
MazF	Toxin (mRNA interferase) component of MazE-MazF module	−
Hfq	RNA chaperone	−

a Category and general description are from UniProt.

b Results from this study.

**FIG 1:**
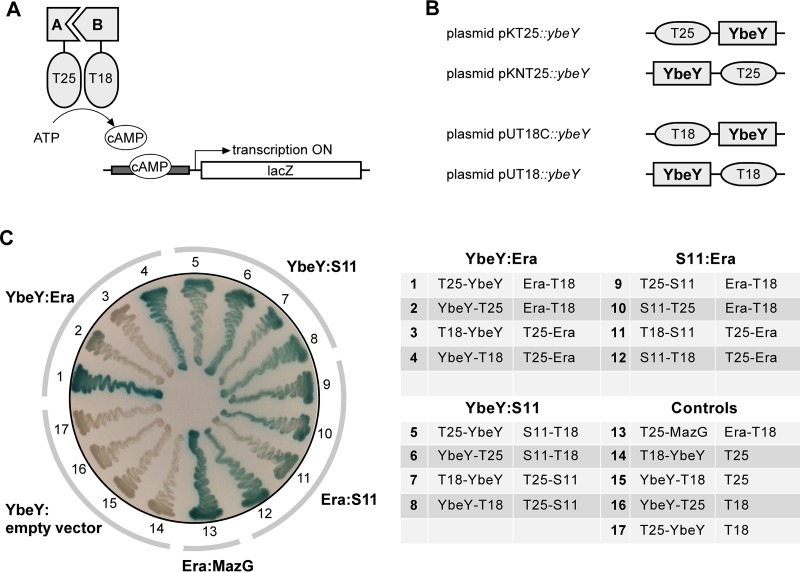
Identification of YbeY interaction partners using B2H analysis. (A) General principle of B2H. Interaction between two candidate proteins (proteins A and B) brings the T25 and T18 fragments of adenylate cyclase together, and the resulting increase in cAMP levels can be monitored directly using a β-galactosidase reporter under control of a cAMP/CAP (catabolite activator protein)-dependent promoter (23, 61). (B) B2H fusion constructs of YbeY. N- and C-terminal fusions were generated using low-copy-number plasmids pKT25 and pKNT25 and high-copy-number plasmids pUT18C and pUT18 (23). (C) YbeY interacts with ribosomal protein S11 and the ribosome-associated GTPase Era. *E. coli* B2H strain BTH101 (23) was transformed with fusion constructs as indicated. (Left) Transformants were streaked on X-Gal indicator plates as described in detail in Materials and Methods and incubated at 30°C for 2 or 3 days. Formation of blue pigment is indicative of a positive interaction between proteins of interest. (Right) Overview of the multiple N- and C-terminal fusions of YbeY, S11, and Era with the Cya fragments T18 and T25 (transformants 1 to 12), including the positive control T25-MazG::Era-T18 (transformant 13) and the negative controls with empty constructs (transformants 14 to 17).

The B2H system used in this study can identify two interacting proteins that are each fused to either the T25 or T18 fragment of the catalytic domain of *Bordetella pertussis* adenylate cyclase (Fig. 1A) (23). When brought together by interaction of the corresponding fusion partners, the T25 and T18 fragments reconstitute the catalytic domain of adenylate cyclase and the increased level of cyclic AMP (cAMP) induces the β-galactosidase reporter, producing blue colonies on agar supplemented with 5-bromo-4-chloro-3-indolyl-β-d-galactopyranoside (X-Gal). Using a standard spotting assay (see Fig. S1A in the supplemental material) based on complementation of a *ybeY* deletion mutant (Δ*ybeY*) (3), we confirmed that expression of *E. coli* YbeY from B2H constructs (N- and C-terminal fusion constructs on low- or high-copy-number plasmids) (Fig. 1B) did not, by and large, affect growth or viability of the bacterial host. B2H experiments were generally carried out in the presence of phenylethyl–d-thiogalactoside (TPEG), a competitive inhibitor of β-galactosidase. TPEG was added to solid media to reduce the level of background signal caused by nonspecific cleavage of X-Gal after prolonged incubation and to increase the level of stringency for positive interactions (Fig. S1B). Every B2H experiment included one positive control and four negative controls. The Era-MazG pair was used as the positive control based on the known interaction between the nucleoside triphosphate (NTP) pyrophosphohydrolase MazG and the essential GTPase Era (28). The four YbeY constructs (in combination with empty vectors) were used separately as negative controls.

### Identification of ribosomal protein S11 and the ribosome-associated GTPase Era as interaction partners of YbeY.

The first set of potential interaction partners of YbeY included a selection of ribosomal proteins and the ribosome-associated GTPase Era (Table 1; see Fig. S2A in the supplemental material). This selection was based on our current understanding of YbeY’s role in 16S rRNA maturation and ribosome quality control. The GTPase Era binds to the pre-30S ribosomal subunit close to the 3′ terminus of 16S rRNA. It has been proposed that Era acts as a chaperone for maturation of 16S rRNA possibly guiding one or more RNases in order to process the 3′ terminus. In doing so, Era serves as a checkpoint for maturation of the 30S subunit (29). The ribosomal protein S1 will subsequently bind in the pocket first occupied by Era, marking the completion of the ribosome assembly process. The Era binding site is located in the cleft region between the head and platform of the 30S subunit interacting with ribosomal proteins S2, S7, S11, and S18 (30). Using B2H, we observed an interaction between YbeY and S11 as well as Era (Fig. 1C; see Fig. S2A and S2B in the supplemental material), but not with ribosomal proteins S1, S2, S7, and S18 (Fig. S2A). The interaction of YbeY and S11 was strong for all four B2H plasmid combinations that were tested. In contrast, only two of the four plasmid combinations showed a positive interaction between YbeY and Era, indicating that the orientation of the respective fusion tags together with intracellular levels of the fusion proteins might play a role in identifying interactors. Era also interacts with S11, hence exposing a three-way association between YbeY, S11, and Era.

### Ribosomal protein S11 coimmunoprecipitates with YbeY.

In an independent approach to identifying interaction partners of YbeY, we used an *E. coli* strain expressing a C-terminally FLAG-tagged YbeY from the chromosome (3). Proteolytic digestion followed by mass spectrometric analysis revealed that YbeY pulled down ribosomal proteins S11, S7, and L6 and ATP synthase subunit B (see Fig. S3 in the supplemental material). These results are consistent with our B2H finding that YbeY interacts directly with the ribosome *in vivo* with S11 as one of the key interaction points.

Although S7 had tested negative in our B2H experiments, we cannot rule out the possibility that the location of the T25 and T18 fragments of adenylate cyclase used in the respective N- and C-terminal B2H fusion constructs was unfavorable to permit signal detection between YbeY and S7. Alternatively, the pulldown of S7 and L6 could also imply an indirect interaction with YbeY via rRNA or other ribosomal proteins. For example, a previous large-scale pulldown analysis listed S7 and L6 as proteins binding to YbeZ (16), which in turn interacts with YbeY as shown in this study (see below). The significance of the association of YbeY with ATP synthase subunit B is unclear.

### YbeY interaction interface differs between S11 and Era.

**FIG 2:**
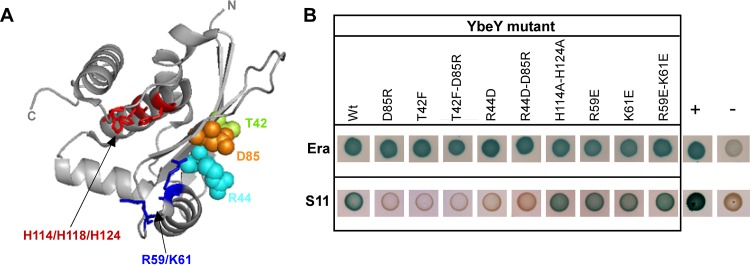
YbeY interaction interface with S11 differs from its interaction interface with Era. (A) Model of YbeY generated in PyMOL (http://www.pymol.org) using PDB accession code 1XM5 (31), showing the positions of conserved residues R59, K61, H114, H118, and H124 in the active site (highlighted in blue and red) and T42 (green), R44 (cyan), and D85 (orange) in the four-stranded beta-sheet. (B) B2H interaction analysis of YbeY mutants with Era and S11, respectively, using T25-YbeY (wild-type [Wt] or mutant), Era-T18, and S11-T18. Experiments were performed in triplicate, and representative spots of BTH101 transformants are shown; positive (T25-MazG::Era-T18) and negative (T25-YbeY::empty T18) controls are indicated by + and −, respectively.

To obtain further insight into YbeY’s mode of interaction with S11 and Era, we sought to identify YbeY’s potential interaction interface. YbeY is a small globular protein (18 kDa) (31) with two well-defined regions that might serve as distinct protein- and/or RNA-interacting interfaces (Fig. 2A). YbeY’s active site consists of a metal-binding histidine triad (H3XH5XH motif) within an alpha-helical channel. Opposite the active site is a four-stranded beta-sheet. We incorporated single and double missense mutations in the T25-YbeY construct that displayed strong interaction with both S11 and Era.

In previous work, we showed that the conserved histidines H114, H118, and H124, as well as arginine R59 and lysine K61 are required for the RNase activity of YbeY (1, 3). Here, we evaluated the roles of these critical residues at the active site by B2H analysis using H114A H124A, R59E, K61E, and R59E K61E YbeY mutants. Similarly, potential interaction with the opposite beta-sheet of YbeY was analyzed by generating D85R, T42F, R44D, D85R T42F, and D85R R44D mutants. These residues are highly conserved across bacterial YbeY proteins and were selected based on their central location in the beta-sheet (Fig. 2A). Charge reversal mutations were chosen over alanine mutations in order to maximally disrupt possible protein-protein interactions.

Complete loss of interaction with S11 was observed for all YbeY mutants in the beta-sheet, but not for mutants in the active site (Fig. 2B), suggesting that S11 is interacting directly with YbeY via highly conserved key residues at the beta-sheet interface. In contrast, no loss of B2H interaction was observed using the multiple YbeY mutants and Era (Fig. 2B), suggesting the possibility that the interaction with Era is indirect or involves other regions of YbeY. It is also possible that the interaction between YbeY and Era involves multiple residues and that mutation in any of them does not alter the binding affinity significantly enough to affect the B2H results.

### YbeY-S11 interaction interface is required for rRNA maturation and stress regulation.

We determined the functional significance of the YbeY-S11 interaction by analyzing the extent of rRNA maturation and cell survival levels under a variety of stress conditions for YbeY mutants with an altered S11 interface. For this, a Δ*ybeY* mutant was transformed with plasmids expressing the respective single and double *ybeY* mutants.

**FIG 3:**
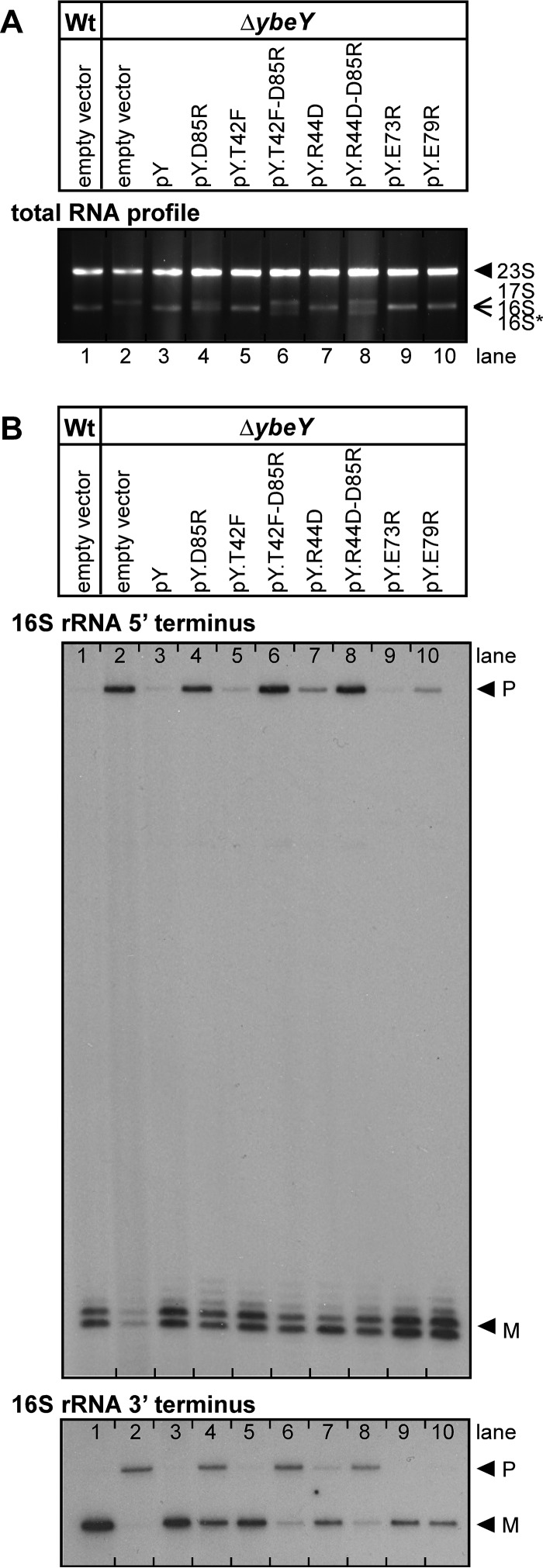
rRNA maturation in *E. coli* Δ*ybeY* mutant complemented by various *ybeY* constructs containing single and double mutations. (A) Analysis of total RNA by agarose gel electrophoresis. The positions of 23S, 17S, 16S, and 16S* rRNAs are indicated to the right of the gel based on their mobility. (B) Mapping of 5′ and 3′ termini of 16S rRNA in total RNA. The positions of bands derived from the precursor (P) and mature (M) forms of 16S rRNA are indicated to the right of the gels. Total RNA was isolated from *E. coli* MC4100 wild-type (Wt) and *ybeY* deletion mutant (Δ*ybeY*) transformed with a plasmid containing the gene for wild-type YbeY and YbeY mutants D85R, T42F, T42F-D85R, R44D, R44D-D85R, respectively; transformants containing the empty vector are shown as controls.

The total rRNA profile for the D85R YbeY mutant showed an intermediate decrease of mature 16S rRNA and a concomitant intermediate increase of 17S precursor levels compared to the wild-type levels (Fig. 3A). Although no clear defect in 16S rRNA maturation was observed for the other single YbeY mutants (T42F and R44D), both double mutants of YbeY (D85R T42F and D85R R44D) showed a stronger accumulation of 17S precursors than the D85R YbeY mutant did. Hence, the T42F and R44D mutations, which are located close to D85 (Fig. 2A), exacerbate the disruption of the protein-interacting interface, demonstrating the relevance of the YbeY-S11 interaction in the rRNA maturation process. Moreover, mapping of the 5′ and 3′ termini of 16S rRNA confirmed the maturation defects of the YbeY interface mutants (Fig. 3B). Intermediate and strongly increased accumulation of precursor with unprocessed 3′ ends was observed for the single and double YbeY mutants, respectively, matching the total rRNA profile. In the case of the 5′ end maturation of 16S rRNA, D85 is mainly required for directing the maturation process, as we were unable to detect any further increase in unprocessed 5′ ends from additionally mutating T42 or R44. This is consistent with the notion that YbeY might act in a different manner or interact with different proteins during the maturation process of the 5′ or 3′ terminus of 16S rRNA.

**FIG 4:**
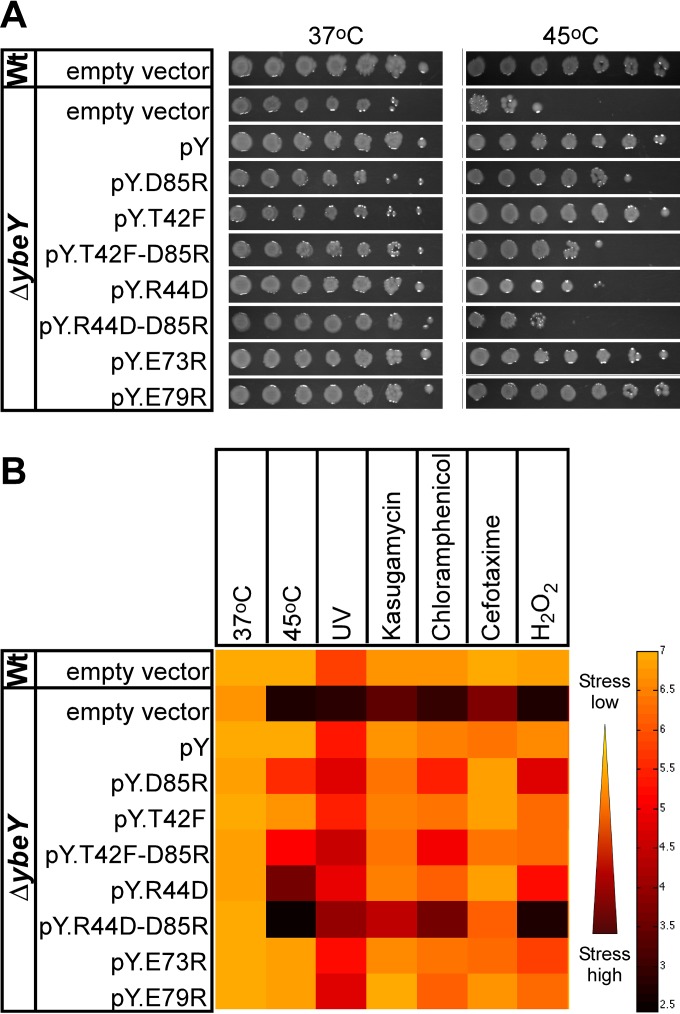
YbeY protein interactions are important in stress regulation. *E. coli* Δ*ybeY* complemented by various *ybeY* single and double mutants is sensitive to various stresses. (A) Heat stress. Wild-type (Wt) and Δ*ybeY* cells complemented with empty pBR322, pBR322-*ybeY* (pY), pY_T42F, pY_R44D, pY_D85R, and pY_T42F-D85R. The cells were grown overnight in LB medium, subsequently diluted to an OD_600_ of 0.1, spotted as a dilution series (1:10) onto LB plates, and incubated overnight at 37°C and 45°C. (B) Overview of stress sensitivity. The cells were incubated overnight at 37°C and 45°C (as shown in panel A), UV (55 J/m^2^), kasugamycin (200 µg/ml), chloramphenicol (2.5 µg/ml), cefotaxime (0.12 µg/ml), and H_2_O_2_ (0.75 mM). The black-to-orange color gradient corresponds to the level of surviving cells, i.e., 10^2.5^ CFU/ml or high stress (black) and 10^7^ CFU/ml or low stress (orange). These results represent the averages from three independent experiments.

The loss of *ybeY* in *E. coli* was previously shown to result in a pleiotropic phenotype that includes a significant sensitivity to physiologically diverse stresses (2, 3). Heat is a major stress factor, as YbeY was first reported to be part of the heat shock response (32) and later found to cause a total lack of mature 16S rRNA in a Δ*ybeY* mutant (1). Interestingly, after heat treatment, the D85R R44D YbeY double mutant shows a reduction in survival that is comparable to that of the *ΔybeY* mutant (Fig. 4A), which is consistent with a similar loss of mature 16S rRNA (Fig. 3A and B). While D85 is more critical in the YbeY-S11 interaction and rRNA maturation than T42 and R44 are, R44 is more critical during heat stress than D85 is. The *ybeY* double mutants show a clear increase in sensitivity compared to the single mutants. A similar phenotypic pattern was detected for the mutants grown under other stress conditions (Fig. 4B). We also observed that YbeY missense mutants with an altered beta-sheet interface displayed an increase in sensitivity to three antibiotics, each with a different mode of action, i.e., kasugamycin, chloramphenicol, and cefotaxime. Moreover, we found that the mutants exhibited a consistent decrease in protection against oxidative and UV stress. Thus, disrupting one or more interaction points between YbeY and S11 is sufficient to obtain phenotypes similar to that of the *ΔybeY* mutant.

Two additional YbeY mutants, E73R and E79R, were included in these experiments. Changing E73 or E79, two conserved glutamic acid residues in the vicinity of D85, to arginine produced YbeY variants that exhibited a wild-type-like rRNA maturation pattern and stress response (Fig. 3 and 4).

### Structural models of YbeY docked to the Era-30S complex are in agreement with *in vivo* data.

To further examine the interaction of YbeY with potential binding partners on the ribosome, we performed computational protein docking studies that are driven first by principles of molecular recognition and assume limited or no knowledge from the B2H data described in this study. The sole assumption was interaction of YbeY with both S11 and Era. Such a “blind docking” approach would provide an independent perspective for protein docking whose results can then be used to compare with the B2H data.

**FIG 5:**
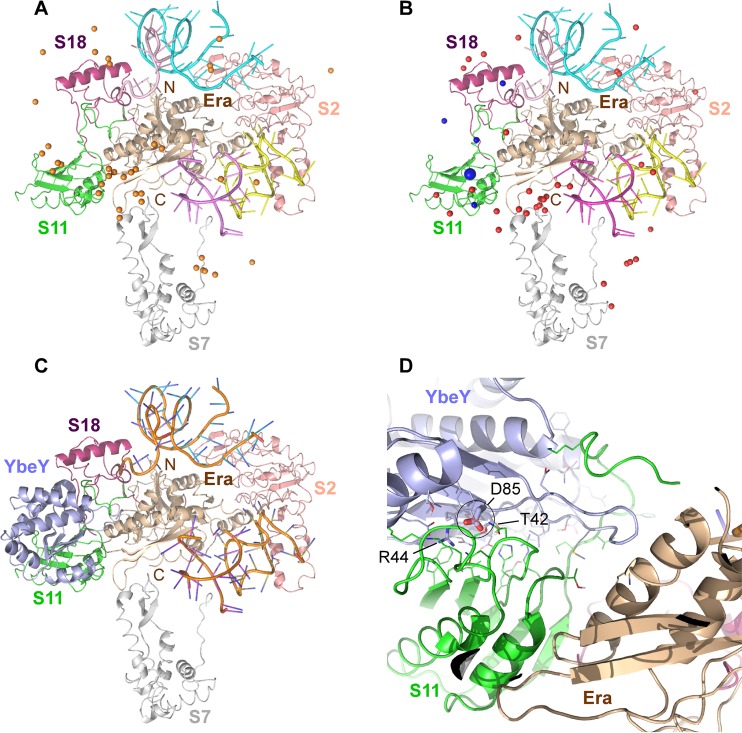
Protein docking model of YbeY binding to the ribosome. YbeY interaction with the 30S ribosomal subunit, as predicted by computational modeling using the cryo-EM structure of *E. coli* Era bound to *T. thermophilus* S1-depleted 30S ribosomes (PDB accession code 1X18). Era (wheat cartoon) and ribosomal proteins S2 (salmon cartoon), S7 (gray cartoon), S11 (green cartoon), and S18 (magenta cartoon) are depicted as indicated. (A) Blind docking model representing possible interactions of YbeY. An ensemble of the top 40 models is shown. The geometric centers of YbeY, approximated by the Cα atoms of V112, are indicated by orange spheres. Portions of RNA helices are shown in cyan, pink, and purple. (B) Same blind docking model as shown in panel A, with the Cγ atoms of D85 indicated as red spheres (within 20 Å from S11) or blue spheres (within 10 Å from S11). (C) One of the top five models showing a possible binding mode of interaction between YbeY (light blue cartoon) and S11. Portions of RNA helices are shown in orange. (D) YbeY-S11 interface from panel C. YbeY residues T42, R44, and D85 (see the larger blue sphere in panel B) are indicated.

The most relevant structure of an Era-30S ribosome complex in the Protein Data Bank (PDB) contains *E. coli* Era and a limited 30S ribosomal subunit from *Thermus thermophilus* (including 16S rRNA along with ribosomal proteins S2, S7, S11, and S18; PDB accession number or code 1X18) (30). After docking *E. coli* YbeY (PDB accession code 1XM5) (31) into an all-atom model of this Era-30S complex, we examined an ensemble of the top 40 models, representing the top 10 models for each of the four binding energy functions used. As shown in Fig. 5A, the geometric centers of YbeY in these models (orange spheres; approximated by the Cα atoms of V112) are found to be on the front side of the Era-30S complex containing rRNA segments, suggesting that the charged rRNA likely contributes to the recognitions between YbeY and the Era-30S complex. In total, 32 (80%) of the 40 models have YbeY close to at least one of S11, S7, and Era. Noticeably, a major cluster of the geometric centers of YbeY models are close to S11: 24 (60%) of all models are within 20 Å (a value chosen based on YbeY’s radius of gyration and insensitivity to the local choice of the atom to represent the geometric center) from S11. Moreover, based on the aforementioned criteria, 13 (32.5%) are close to S7, including 6 close to both S11 and S7, and 9 (22.5%) are close to Era, including 8 close to both S11 and Era. A major binding site of YbeY seems to be close to S11 near its interface with Era; no models supported YbeY interacting with Era’s C-terminal KH domain (on the back side in Fig. 5A), even though the domain appears much more widely accessible for potential interactions compared to the suspected major binding site. These docking results support the notion, based on our B2H results, of S11 and Era as binding partners of YbeY.

To investigate the binding mode of YbeY and the role of D85, we examined D85’s positions with their Cγ atoms as spheres shown in Fig. 5B (red or blue spheres). The D85 residues in these models are near S11; 22 of the 24 models where YbeY’s centers are within 20 Å from S11 also have D85 within 20 Å from S11. Among these models, five models (blue spheres) supported D85 to be close enough (within 10 Å) to S11, consistent with the B2H data showing that mutation of D85 disrupts the binding interface with S11. We chose one of the five models whose D85 (shown by the larger blue sphere in Fig. 5B) interacts with S11 at its structurally stable beta-sheet region. We then further examined the model using our mutagenesis data for YbeY residues T42, R44, and D85. As shown in Fig. 5C, the docked YbeY interacts with S11 and, meanwhile, is in proximity to Era. A closer look (Fig. 5D; rotated from the view in Fig. 5C for better visualization) indicates that the YbeY-S11 interface consists mostly of packed beta-sheets. Packing appears to be driven mostly by hydrophilic forces because interface side chains (shown as sticks) within 3 Å of their possible intermolecular binding partners are mostly charged or polar. In particular, D85 of YbeY was shown to potentially interact with N28 or N29 of S11 with a possible addition of K57 (using *E. coli* numbering for S11). These residues are highly conserved even across relatively distant species like *Mycobacteria*, *Mycoplasma*, and *Helicobacteria*. In addition, consistent with the mutagenesis data for YbeY-S11 B2H interaction, T42 and R44 of YbeY also appear at the binding interface in this model. Interestingly, the tip of a loop region of YbeY (around G77) is within 4 Å from Era K263, indicating a possible interaction that might be weak or transient but does not depend on D85 of YbeY, as the YbeY-Era B2H data suggested earlier.

### Additional YbeY interactors: Der, YbeZ, and SpoT.

Because of the multifaceted function of YbeY, we expanded the set of candidate interactors screened by B2H and tested possible interactions between YbeY and a number of proteins, focusing mostly on ribosome biogenesis, small RNA regulation, and stress response (Table 1; see Fig. S2A in the supplemental material).

### (i) Ribosome biogenesis factors.

The ribosome assembly cofactors KsgA, RsgA, RbfA, and RimM are associated with the 30S subunit and are part of a regulatory network around Era (13). However, in contrast to Era, none of the four assembly cofactors was shown to physically interact with YbeY in our B2H analysis. In addition, we studied YbeY’s interaction with two other highly conserved GTPases, Der (double-Era-like GTPase) and ObgE, showing a positive interaction with Der but not with ObgE (see Fig. S2A in the supplemental material).

### (ii) RNase R, PNPase.

We have previously shown that RNase R, one of the three major 3′-to-5′ processing exoribonucleases in *E. coli*, together with YbeY is required for degradation of immature or misprocessed 70S ribosomes (1). The proposed model for this ribosome quality control suggests that YbeY likely initiates the 70S degradation by making endonucleolytic cuts, which are then exposed to RNase R that can unwind the rRNA and continues to degrade the ribosome exonucleolytically, possibly assisted further by YbeY. Not surprisingly, a *ΔybeY Δrnr* double mutant presents a severe growth phenotype accompanied by a pronounced 16S rRNA maturation defect (3). Although RNase R is also recruited to the ribosome via ribosomal protein S12 (33–35) and despite the strong functional relationship between YbeY and RNase R, no B2H interaction was detected. Likewise, although our previous studies revealed a strong functional interaction between YbeY and polynucleotide phosphorylase (PNPase), similarly characterized by deficiencies in rRNA maturation in a *ΔybeY Δpnp* double mutant (3), we were unable to observe a direct B2H interaction between these two proteins.

### (iii) Hfq and the RNA degradosome.

We selected the RNA chaperone Hfq as another possible candidate interactor based on several studies that showed YbeY to play a role in sRNA regulation in bacteria (2, 7–9). Hfq is the general sRNA chaperone in bacteria and is part of the RNase E degradosome that includes RNase E, PNPase, and the DEAD box RNA helicase RhlB (36–38). The lack of a B2H interaction with Hfq, RNase E, PNPase, or RhlB indicates that YbeY acts independently but alongside these proteins in sRNA regulation.

### (iv) MazEF.

Another nuclease of interest is MazF. MazF is the nuclease component of the toxin-antitoxin module MazEF, which under certain stress conditions removes the 3′ end of 16S rRNA containing the anti-Shine-Dalgarno site to produce a subpopulation of ribosomes, and concomitantly removes the 5′ untranslated regions (5′ UTR) of specific mRNAs, thereby generating leaderless mRNA (39). Nevertheless, since we could not detect an interaction between YbeY and MazE or MazF, we could not show an immediate link between YbeY and this intriguing mechanism of stress ribosomes translating leaderless mRNAs.

### (v) YbeZ.

YbeZ is a protein of unknown function with a nucleoside triphosphate hydrolase domain and belongs to the subfamily of PhoH proteins. In *E. coli*, *ybeZ* is located upstream of *ybeY*, forming the *ybeZY* operon. Our B2H analysis showed YbeZ interacting strongly with YbeY (see Fig. S2A in the supplemental material).

### (vi) Stringent response.

The stringent response regulates the overall bacterial stress response under unfavorable growth conditions via the alarmone guanosine (penta)tetraphosphate (p)ppGpp (40, 41). Since YbeY was shown to be important for survival under a wide range of stress conditions (2, 3), and the main regulators of the stringent response are, like YbeY, closely associated with the ribosome, we examined whether YbeY interacts with SpoT, RelA, DksA, and BipA. Only SpoT tested positive in our experiments (see Fig. S2A in the supplemental material).

### (vii) Initiation factors IF2 and IF3.

Last, a possible interaction of YbeY with translation initiation factor IF2 or IF3 was also analyzed. In earlier work, the binding of both IF2 and IF3 to the 30S ribosomal subunit was found to be affected in a *ΔybeY* mutant (3). IF2 levels were shown to be decreased, while IF3 levels were markedly increased, consistent with the idea that IF3 acts as an anti-association factor, ensuring that immature or improperly processed 30S subunits found in the *ΔybeY* mutant were kept from assembly into 70S ribosomes. However, no interaction was observed between YbeY and either of these factors using B2H.

## DISCUSSION

We have shown for the first time that YbeY interacts specifically with several proteins associated with ribosome maturation and stress regulation, i.e., S11, Era, Der, YbeZ, and SpoT (Fig. 1 and Table 1), observations which suggest that YbeY is recruited to the ribosome. Moreover, we have identified key residues of YbeY distant from its catalytic site that are critical for its interaction with the ribosomal protein S11 (Fig. 2A). Finally, we have combined these insights with *in silico* protein docking techniques to propose a model for how YbeY binds to the 30S ribosomal subunit to enable the final processing events of the 3′ end of 16S rRNA.

### YbeY’s interaction with ribosomal protein S11 and ribosome maturation factor Era.

YbeY plays a critical role in *E. coli*’s stress response, translational fidelity, ribosome biogenesis, and activity (3). All three rRNAs, which are originally transcribed with extensive 5′ and 3′ trailing sequences, require YbeY for maturation. At 45°C, an *E. coli* ΔybeY mutant is essentially depleted of fully matured 16S rRNA. YbeY is the first and, so far, the only endoribonuclease to be implicated in the processing of the 3′ end of 16S rRNA, which is critical for initiation of mRNA translation (1, 3). Also, YbeY and RNase R were reported to act together in a previously unrecognized late-stage ribosome quality control system that removes defective 70S ribosomes (1). While YbeY’s cellular role is well described, a detailed understanding of its mode of action is missing. Using a B2H analysis that focused on a small number of potential interactors based on YbeY’s cellular functions, we identified key interacting proteins obtaining new insights into its function.

A primary potential interaction partner of YbeY was Era, which serves as a chaperone for the maturation of 16S rRNA, possibly guiding one or more RNases to process the 3′ terminus (29). In Era-depleted cells, 16S rRNA precursors accumulate, and the levels of individual 30S and 50S subunits increase relative to the levels of 70S ribosomes (42). The observed B2H interaction between YbeY and Era (Fig. 1C; see Fig. S2B in the supplemental material) suggests that both proteins are temporally coming together, close to the 3′ processing site of 16S rRNA.

During ribosome assembly, Era binds temporarily to the pre-30S subunit (29). Cryo-electron microscopy (cryo-EM) analysis has shown that its binding site is comprised of rRNA segments, including the 3′ end of 16S rRNA and several ribosomal proteins, i.e., S2, S7, S11, and S18 (30). We observed a strong B2H interaction between S11 and YbeY (Fig. 1C) and identified critical interacting residues in YbeY’s beta-sheet (for more details, see below). Therefore, YbeY is most likely recruited to the ribosome via interactions with both Era and S11. This strong interaction with S11 was independently confirmed by pulldown experiments (see Fig. S3 in the supplemental material) and corroborated by blind protein docking models (Fig. 5). The S11 interaction with YbeY has also been listed in system-wide Y2H studies of *Synechocystis* sp. strain PCC6803 (43) and *Helicobacter pylori* (44), suggesting that it is evolutionarily conserved.

### A model for how YbeY’s interaction with S11 and Era guides the final processing events of the 3′ end of 16S rRNA.

**FIG 6:**
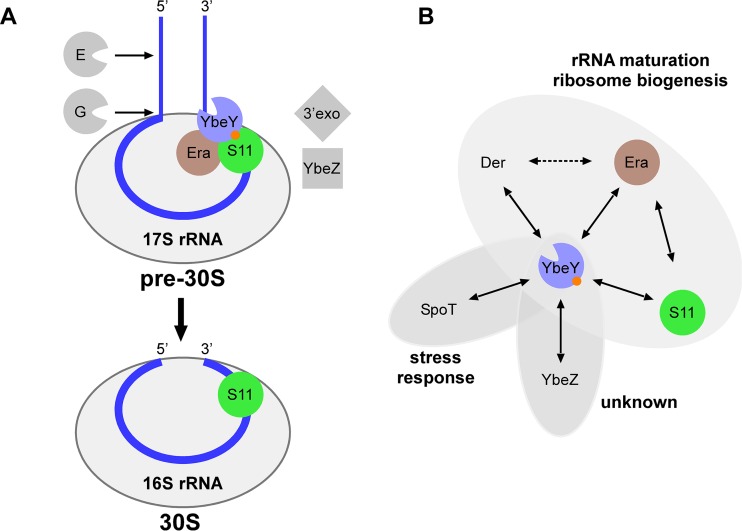
(A) Model for 16S rRNA maturation in *E. coli*. Maturation of the 5′ end of 16S rRNA requires the sequential action of RNases E and G (15). Our work suggests that YbeY is required for maturation of the 3′ end (1, 3). To do so, YbeY is recruited to the ribosome through interactions with ribosomal protein S11 and the GTPase Era (this work; the orange dot indicates D85 in YbeY’s beta-sheet) and cleaves the 17S rRNA precursor endonucleolytically at or near the final 3′ end maturation site. The final maturation steps could be carried out by 3′ exonucleases (3’exo) such as RNase R or PNPase (45) and/or the predicted phosphohydrolase YbeZ, one of the proteins found to interact with YbeY in this work. (B) The YbeY interaction network based on B2H.

The observed B2H interaction of YbeY to S11 and Era is also in agreement with previous studies on the maturation process of the critical 3′ end of 16S rRNA. We recently suggested that YbeY cleaves the 17S rRNA precursor endonucleolytically at or near the final 3′ end maturation site, generating a 3′ phosphate (1). To produce the mature 16S rRNA 3′ end with a 3′ hydroxyl, the final maturation steps could be carried out by 3′ exonucleases such as RNase R, PNPase (45), and/or the predicted phosphohydrolase YbeZ, one of the proteins found to interact with YbeY in this work. However, if YbeY acts directly on the 17S rRNA precursor, then some other factor(s) would be required to restrict YbeY’s otherwise nonspecific endonucleolytic activity (1) to the maturation site of 16S rRNA. Considering a 30S preribosomal intermediate as the substrate, the GTPase Era and nearby ribosomal proteins are plausible candidates for guiding YbeY’s activity (12–14). Tu et al. showed how Era can bind to pre-16S rRNA, locking the pre-30S subunit in a conformation that is not favorable for association with the 50S ribosomal subunit and that may facilitate the final processing of 16S rRNA by RNase E, RNase G, and an unknown RNase (29, 46). They proposed that Era acts as a chaperone for processing and maturation of 16S rRNA, as Era releases 30S subunits with mature 16S rRNA after GTP hydrolysis. We propose that YbeY can serve as the unknown RNase in the latter model because nucleotides adjacent to the mature 16S rRNA 3′ end are freely protruding out of the Era-16S rRNA complex and are therefore accessible to an endonuclease like YbeY (Fig. 6A).

To further substantiate the above models, we attempted to predict atomic-level details of YbeY’s interaction with the 30S subunit by computational protein docking. We began with the cryo-EM structure of a *T. thermophilus* 30S-Era complex (S1-depleted) into which the X-ray structure of *E. coli* Era was fitted (30). This structure shows how the C-terminal KH domain of Era can interact with the 3′ end of 16S rRNA. In addition to the rRNA interaction, Era’s C-terminal domain also interacts with the ribosomal proteins S7 and S11, respectively, whereas S18 interacts with the N-terminal domain of Era. After first generating an all-atom structural model for the 30S-Era complex, we docked *E. coli* YbeY to this 30S-Era structure in a blind manner to be as unbiased as possible. Interestingly, a significant majority of the top prediction models shows YbeY to be in close vicinity to Era and S11, which are located between the cleft and platform of the 30S subunit (Fig. 5A). Therefore, the blind docking models are overall in agreement with our experimental data.

To understand in more detail YbeY’s interaction with Era and S11, we analyzed the B2H interaction using several YbeY mutants, including enzymatically impaired mutants (1, 3) and mutants with mutations in the beta-sheet, which is located opposite the enzymatic site (Fig. 2A). We observed a loss of B2H interaction with S11 in the case of the YbeY beta-sheet mutants (D85R, T42F, and R44D) but did not see reduced B2H signal with Era. These cell-based results are also in agreement with the *in silico* results, as several models could be identified with YbeY D85R within 10 Å of S11 (Fig. 5B). We show one representative docking model in detail to illustrate how the three residues of YbeY can potentially interact with S11 (Fig. 5C and D). Also, in this docking model, YbeY covers S11 rather extensively while interacting only modestly with Era. Such interaction fits with the strong B2H signal observed between YbeY-S11 and the weaker YbeY-Era signal (see Fig. S2B in the supplemental material). Moreover, the interaction of the YbeY beta-sheet with S11 allows YbeY’s catalytic site to be accessible to rRNA in the vicinity of the 16S rRNA 3′ end maturation site. Therefore, the binding mode of YbeY that we suggest is consistent with our earlier proposal for YbeY’s role in 16S rRNA maturation. Our results support the hypothesis that YbeY acts as the so far unknown RNase involved in the final maturation events of 16S rRNA, with Era and S11 guiding its otherwise nonspecific endonucleolytic activity. Finally, the D85R mutation in YbeY results in partially defective 16S rRNA 3′ end maturation (Fig. 3) and pleiotropic stress phenotype (Fig. 4), both of which are exacerbated in the case of the D85R T42F and D85R R44D double mutants, further indicating that YbeY’s interaction with S11 is critical to correctly guide its 16S rRNA maturation function.

### Potential implications of YbeY’s interactions with Der, YbeZ, and SpoT.

The possible interaction of YbeY with two other ribosome-associated GTPases, i.e., Der and ObgE, was also evaluated. These essential GTPases are critical molecular switches in regulating ribosome maturation and assembly. Although they are associated mainly with the 50S subunit, depletion of each results in an increase of individual ribosomal subunits and rRNA precursors, reminiscent of the ribosomal phenotype of the *ΔybeY* mutant (3, 47, 48). The GTPase Der, structurally similar to Era (containing two GTP binding domains instead of one like Era), binds to 30S subunits only when bound to GDP (49) and was previously shown in *Salmonella enterica* serotype Typhimurium to interact with ribosomal proteins S7 and S9, which are close to the Era binding site in 16S rRNA (30, 50). We detected a positive B2H interaction between YbeY and Der in this study. Further investigation is needed to determine whether the interaction between YbeY and Der plays a role in ribosome biogenesis. In contrast to Era and Der, no B2H interaction between YbeY and the ribosome-associated GTPase ObgE was observed.

The cooccurrence of genes encoding YbeZ and YbeY in a single operon is highly conserved among bacteria, suggesting a functional connection between these proteins. Although its function is still unknown, YbeZ may be involved in the final steps of 16S rRNA maturation as described above. The B2H interaction that we observed between YbeY and YbeZ could support this hypothesis (16).

The loss of YbeY was previously shown to severely impair stress tolerance, which was mostly attributed to defects in translational efficiency and accuracy (2, 3). Here, we observed for the first time a direct connection between YbeY and the stringent response. The stringent response is activated under various unfavorable growth conditions and coordinated by the alarmone (p)ppGpp that induces a general reprogramming of gene regulation diverting available resources to allow adaptation to a nongrowing state (40, 51). *E. coli* has two closely related enzymes, SpoT and RelA, for the synthesis and degradation of (p)ppGpp. SpoT can both synthesize and degrade the alarmone, whereas RelA can only produce it. We considered both SpoT and RelA as potential YbeY interactors because both regulators are associated with the ribosome and mutants defective in the stringent response show a pleiotropic phenotype under stress conditions similar to that of the Δ*ybeY* mutant. We also included the transcription factor DksA, which is principally known to regulate synergistically with (p)ppGpp (52), and the GTPase BipA (53), which when bound to (p)ppGpp specifically associates with 30S subunits (54). We observed a strong B2H interaction between YbeY and SpoT, but not RelA, DskA, or BipA. The exact nature of YbeY’s interaction with SpoT is unclear, since SpoT is mainly associated with the 50S subunit. However, an AP-MS study also showed interaction between SpoT and S11 (16), suggesting S11 as a possible link between SpoT and YbeY.

### Potential implications of apparent negative B2H interactions between YbeY and other proteins involved in RNA metabolism.

On the basis of previous work on YbeY, we also tested possible interactions between YbeY and numerous ribosome assembly cofactors (KsgA, RsgA, RbfA, and RimM), RNase R, PNPase, RNase E, helicase RhlB, Hfq, MazEF, and initiation factors IF2 and IF3, respectively (Table 1; see Fig. S2A in the supplemental material), all of which tested negative. It is important to note that our B2H interaction analyses between YbeY and ribosomal protein S11 and Era were carried out with a full set of plasmid constructs, using both N- and C-terminally tagged variants on high- or low-copy plasmids, respectively (Fig. 1). For our analyses of “other” possible candidates, we focused on the combination of plasmids T25-YbeY with x-T18 (x representing any of the other possible interactors). Therefore, we cannot exclude the possibility that some of the above candidate proteins might still interact with YbeY if the orientation of the T25 and T18 fragments or the copy number of the respective plasmids is important for a clear B2H signal. These apparent negative B2H data, although possibly affected by tagging or copy number effects, may indeed represent the absence of interaction between two proteins or interactions that are too weak or highly transient. Most of our negative YbeY interactors have also been part of studies representing global analyses of the *E. coli* protein-protein interactions network (16–18, 20, 21). These studies have used completely different approaches (AP-MS, GI, proteomic/genomic context) and observed a similar lack of interaction with YbeY, consistent with the negative B2H interactions in this study. It is also interesting to point out that the observed S11 interaction with YbeY is among the more than 11,000 positive and negative interactions listed in an earlier system-wide AP-MS analysis in *E. coli* using YbeY as the bait (17), yet the same study failed to detect interactions between YbeY and Era, Der, YbeZ, or SpoT. Similarly, another global network study with more than 6,000 listed interactions detected the protein-protein interaction between YbeY and its operon partner YbeZ but none of the other aforementioned YbeY interactions (16). As one might expect, the different approaches to study large-scale protein-protein interactions *in vivo* have their inherent advantages and disadvantages or limitations. Nonetheless, by focusing on a select number of candidate interaction partners based on our current understanding of YbeY’s cellular role, we were able to identify novel YbeY protein-protein interactions using B2H (Fig. 6B).

### Concluding remarks.

This study provides new mechanistic insights into YbeY’s multifaceted cellular functions and opens up a number of future directions: the identification of S11, Era, Der, YbeZ, and SpoT as YbeY interaction partner requires further analysis. Besides YbeY’s interaction with S11 and Era that, according to our observations, localizes most likely to the ribosome, the question remains whether the interactions between YbeY and Der, YbeZ, and SpoT are also ribosome associated and, if so, under which conditions these interactions occur. Likewise, the characterization of YbeZ’s biochemical function needs to be addressed. Understanding the functional relationship between both operon partners is an important piece of the YbeY puzzle. Additional functional interactors with YbeY could be discovered by expanding the set of B2H constructs and combinations, as well as by a large-scale gene interaction analysis, which would involve the construction of multiple *ybeY* double mutants. Still, the most intriguing question is to further dissect YbeY’s role in ribosome maturation by direct biochemical experiments to validate our hypothesis that YbeY along with accompanying exoribonucleases processes the 16S rRNA 3′ terminus guided by the essential ribosomal assembly factor Era and ribosomal protein S11.

## MATERIALS AND METHODS

### General.

DNA and RNA-DNA chimeric oligonucleotides were obtained from IDT or Eurofins MWG Operon. DNA manipulations were performed by the methods of Sambrook and Russell (55), and cloning products were sequence verified.

### Plasmid construction for complementation assays.

The complementation plasmids expressing YbeY of *E. coli* MC4100 with single or double missense mutations (T42F, R44D, E73R, E79R, D85R, T42F-D85R, R44D-D85R) were constructed by QuikChange site-directed mutagenesis using pBR322-*ybeY* (3) as the template, named pY, which contains *ybeY* downstream of the tetracycline promoter. PCR was performed by PfuTurbo DNA polymerase at 95°C for 30 s and 18 cycles, with 1 cycle consisting of 95°C for 30 s, 55°C for 1 min, and 68°C for 5 min. The constructed plasmids were first transformed into *E. coli* XL1-Blue competent cells (Agilent) after DpnI digestion of template plasmids and subsequently transformed into *E. coli* MC4100 *ΔybeY* (3). All constructs were verified by sequencing.

### Bacterial strains and growth conditions for complementation assays.

The bacterial strains used for this work are wild-type *E. coli* MC4100 (56), *E. coli* MC4100 *ΔybeY* (3), and *E. coli* MC4100 *ΔybeY* complemented with pY, pY_T42F, pY_R44D, pY_E73R, pY_E79R, pY_D85R, pY_T42F-D85R, pY_R44D-D85R (this work).

Strains were grown aerobically in LB medium at 37°C, except for heat shock and protein interaction experiments, where strains were grown at 45°C and 30°C, respectively (see below for more detail). The following antibiotics and concentrations were used for strain selection: ampicillin (50 µg/ml^−1^) and kanamycin (20 µg/ml^−1^).

### Stress phenotypic analysis.

The effects of the YbeY missense mutations on stress sensitivity were determined by spotting serially diluted (1:10; starting at an optical density at 600 nm [OD_600_] of 0.1) cultures, grown overnight in LB medium and subsequently washed with 0.85% saline solution, onto LB plates containing various antibiotics or 0.75 mM H_2_O_2_, as indicated. For UV survival, cultures of the *E. coli* strains were spotted onto LB plates, irradiated with a UV dose of 55 J/m^2^ using a G15T8 UV lamp (GE) at 254 nm, and then incubated in the dark. For heat stress, plates were incubated overnight at 45°C.

### Isolation and analysis of RNA.

Total RNA was isolated from cultures grown first to an OD_600_ of 0.2 to 0.3 in LB at 37°C and subsequently incubated at 45°C for 30 min using E.Z.N.A. total RNA kit I (Omega Bio-Tek) according to the manufacturer’s protocol. Total rRNA profiles were analyzed by Synergel/agarose gel electrophoresis as described previously (57).

### Mapping of 5′ and 3′ termini of rRNA and Northern blot analysis of RNA.

Mapping of the 5′ and 3′ termini of rRNA and Northern blot analysis of RNA were carried out essentially as described before (2). To map the 5′ terminus of 16S rRNA, primer extension assays were performed using Superscript II reverse transcriptase (Invitrogen). The 3′ terminus of 16S rRNA was mapped by site-specific RNase H cleavage assay as described by Li and colleagues (58–60) with minor modifications (2), followed by Northern hybridization using probes specific for the mature 3′ terminus of 16S rRNA.

### BACTH plasmid construction.

To construct the plasmids for the bacterial adenylate cyclase two-hybrid (BACTH) analysis, the coding sequences of the proteins of interest were amplified by PCR using chromosomal DNA of *E. coli* MC4100. The PCR products were digested with XbaI or BamHI (5′ end) and SmaI or KpnI (3′ end), and subsequent fragments were cloned into the BACTH plasmids pUT18, pUT18C, pKT25, and pKNT25 (23, 61), which were digested with the same enzymes. The plasmids pUT18 (high copy) and pKTNT25 (low copy) were used to obtain N-terminal fusions with the T18 and T25 fragments of adenylate cyclase Cya from *B. pertussis*, respectively. Similarly, C-terminal fusions were constructed using the plasmids pUT18C and pKT25. The N-terminal fusions were designated “protein name”-T18 and “protein name”-T25, whereas the C-terminal fusions were designated T18-“protein name” and T25-“protein name” (Fig. 1B). All constructs were verified by sequencing.

### BACTH assay.

Protein-protein interactions were identified using a BACTH system, which is based on the interaction-mediated reconstruction of Cya from *B. pertussis* in the Cya-deficient *E. coli* reporter strain BTH101 (23, 61). Cya consists of two fragments, T18 and T25, which are inactive when separated but functional upon interaction between protein fusions. Interaction was detected by monitoring the β-galactosidase activity in strain BTH101, which expresses LacZ under positive control of a cAMP/CAP (catabolite activator protein)-dependent promoter. Competent BTH101 cells were cotransformed with BACTH plasmids and screened on LB agar (1.2%) plates containing carbenicillin (55 µg/ml) and kanamycin (55 µg/ml). The plates were incubated overnight at 30°C. Three single colonies were subsequently used to inoculate three LB cultures containing carbenicillin (55 µg/ml) and kanamycin (55 µg/ml).

For blue/white screening, 5-µl spots of each overnight culture, incubated at 30°C, were added to LB agar containing carbenicillin (55 µg/ml), kanamycin (55 µg/ml), X-Gal (50 to 75 µg/ml), isopropyl-β-d-thiogalactopyranoside (IPTG) (0.5 to 0.75 mM), and TPEG (93.75 to 125 µg/ml). The latter plates were also used for streaking out individual colonies. The color of the spots and streaks was observed after 3 days at 30°C. The plasmids T25-MazG and Era-T18 served as positive controls for the cotransformations. Cotransformations of T18 or T25 fusions with YbeY or Era together with a nonfused T25 or T18 fragment represented the negative controls.

For the liquid culture assay, cotransformed overnight cultures were used for inoculation, and cultures were grown in LB containing carbenicillin (55 µg/ml), kanamycin (55 µg/ml), and IPTG (0.5 mM). The β-galactosidase activity was measured using the Beta-Glo assay system (Promega) according to the manufacturer’s protocol on a Sirius tube luminometer (Berthold Detection Systems). Relative β-galactosidase activities (given in relative luminescence units [RLU]) were normalized to cell density at the time of harvest.

### Coimmunoprecipitation and peptide identification.

*E. coli* strain MC4100 expressing either native YbeY or a C-terminal FLAG-tagged YbeY (3) was grown to mid-exponential phase in LB at 37°C. Cultures were concentrated and then lysed using Bug Buster native lysis buffer (EMD Biosciences). Lysates were clarified by centrifugation and incubated with an M2 anti-FLAG antibody conjugated to Sepharose beads (Sigma) at 4°C for 3 h. Beads were pelleted and washed three times. Protein sample buffer was added directly to the beads, samples were boiled and then loaded on a 4 to 12% SDS-polyacrylamide gel for protein separation. Proteins were stained using Coomassie brilliant blue dye. Bands 1 and 2 (see Fig. S3 in the supplemental material) were excised from the gel and submitted for matrix-assisted laser desorption ionization−time of flight (MALDI-TOF) mass spectrometry peptide identification at the MIT Biopolymers Laboratory.

### Structural modeling of YbeY-protein interactions.

A cryo-EM structure of *E. coli* Era bound to *Thermus thermophilus* S1-depleted 30S subunit was downloaded from the Protein Data Bank (PDB accession code 1X18). The structure contains only coordinates of phosphorous atoms on RNA and central carbon atoms on proteins. Thus, an all-atom model was initialized using 1X18 as a template, optimized using 1,000 iterations of variable target function method with conjugate gradients, and then refined using molecular dynamics with simulated annealing, all of which was achieved with the program MODELLER (62). A crystal structure of *E. coli* YbeY (PDB accession code 1XM5) was rigidly docked to the all-atom model of limited 30S-Era structure using a webserver ClusPro (63). The top 10 models were collected for each of four sets of differently weighted sum of energy terms such as electrostatics, van der Waals, and knowledge-based statistical potentials derived from known protein-protein interactions. These top predictions (40 in total) were visualized and analyzed using the program PyMOL (http://www.pymol.org).

## SUPPLEMENTAL MATERIAL

Figure S1  YbeY constructs used for B2H analysis. (A) Spotting assay of *E. coli* MC4100 Δ*ybeY* transformed with the four B2H fusion constructs of YbeY used in this study (shown in Fig. 1B). Overexpression of fusion constructs does not substantially affect cell growth or viability. Only the high-copy-number plasmids expressing YbeY fused to the T18 fragment showed a slight reduction in growth. (B) Addition of TPEG to indicator plates reduced the formation of nonspecific background synthesis of β-galactosidase after prolonged incubation. Transformants of the *E. coli* B2H strain BTH101 (top, positive interaction; bottom, negative control) were streaked on plates containing 0, 12.5, 31.3, 62.5, or 93 µg/ml of TPEG and incubated at 30°C for 2 or 3 days. The highest TPEG concentration gave the cleanest background and was subsequently used in all experiments. Download Figure S1, TIF file, 2.2 MB

Figure S2  B2H analysis of interactions between YbeY and selected proteins of interest. (A) B2H analysis of YbeY (T25-YbeY) and selected candidate interaction partners (x-T18). YbeY shows interaction with the ribosome-associated GTPases Era and Der, ribosomal protein S11, the stringent response regulator SpoT, and YbeZ. Experiments were performed in triplicate, and representative streaks of BTH101 transformants are shown. The positive and negative controls were T25-MazG::Era-T18 and T25-YbeY::empty (T18), respectively. (B) Analysis of interaction between YbeY and S11 or Era in liquid culture using the T25-YbeY and x-T18 constructs. β-Galactosidase activity was measured over the course of 72 h and is given in relative luminescence units (RLU) normalized over cell density (OD_600_). Averages from six transformants are shown. Download Figure S2, TIF file, 1.5 MB

Figure S3  YbeY pulldown analysis. FLAG-tagged YbeY was immunoprecipitated from an *E. coli* MC4100 whole-cell lysate. Immunoprecipitates were separated by SDS-PAGE. Proteins in bands 1 and 2 were identified by MALDI-TOF mass spectrometry. Band 1 (b1) contained ribosomal proteins S7, S11, and L6. Band 2 (b2) contained subunit B of ATP synthase. An MC4100 strain carrying a non-FLAG-tagged YbeY was used as the control. The positions of size markers are shown in kilodaltons. Download Figure S3, TIF file, 1 MB

## References

[1] Jacob AI, Köhrer C, Davies BW, RajBhandary UL, Walker GC 2013 Conserved bacterial RNase YbeY plays key roles in 70S ribosome quality control and 16S rRNA maturation. Mol Cell 49:427–438. doi:10.1016/j.molcel.2012.11.025. 23273979 PMC3570609

[2] Vercruysse M, Köhrer C, Davies BW, Arnold MF, Mekalanos JJ, RajBhandary UL, Walker GC 2014 The highly conserved bacterial RNase YbeY is essential in *Vibrio cholerae*, playing a critical role in virulence, stress regulation, and RNA processing. PLoS Pathog 10:e1004175. doi:10.1371/journal.ppat.1004175. 24901994 PMC4047096

[3] Davies BW, Köhrer C, Jacob AI, Simmons LA, Zhu J, Aleman LM, RajBhandary UL, Walker GC 2010 Role of *Escherichia coli* YbeY, a highly conserved protein, in rRNA processing. Mol Microbiol 78:506–518. doi:10.1111/j.1365-2958.2010.07351.x. 20807199 PMC2959132

[4] Liu J, Zhou W, Liu G, Yang C, Sun Y, Wu W, Cao S, Wang C, Hai G, Wang Z, Bock R, Huang J, Cheng Y 2015 The conserved endoribonuclease YbeY is required for chloroplast ribosomal RNA processing in *Arabidopsis*. Plant Physiol 168:205–221. doi:10.1104/pp.114.255000. 25810095 PMC4424013

[5] Davies BW, Walker GC 2008 A highly conserved protein of unknown function is required by *Sinorhizobium meliloti* for symbiosis and environmental stress protection. J Bacteriol 190:1118–1123. doi:10.1128/JB.01521-07. 18055601 PMC2223554

[6] Ohyama H, Sakai T, Agari Y, Fukui K, Nakagawa N, Shinkai A, Masui R, Kuramitsu S 2014 The role of ribonucleases in regulating global mRNA levels in the model organism *Thermus thermophilus* HB8. BMC Genomics 15:386. doi:10.1186/1471-2164-15-386. 24884843 PMC4229858

[7] Pandey SP, Minesinger BK, Kumar J, Walker GC 2011 A highly conserved protein of unknown function in *Sinorhizobium meliloti* affects sRNA regulation similar to Hfq. Nucleic Acids Res 39:4691–4708. doi:10.1093/nar/gkr060. 21325267 PMC3113577

[8] Pandey SP, Winkler JA, Li H, Camacho DM, Collins JJ, Walker GC 2014 Central role for RNase YbeY in Hfq-dependent and Hfq-independent small-RNA regulation in bacteria. BMC Genomics 15:121. doi:10.1186/1471-2164-15-121. 24511998 PMC3933206

[9] Bobrovskyy M, Vanderpool CK, Richards GR 2015 Small RNAs regulate primary and secondary metabolism in Gram-negative bacteria. Microbiol Spectr 3(3):MBP-0009-2014. doi:10.1128/microbiolspec.MBP-0009-2014. 26185078

[10] Leskinen K, Varjosalo M, Skurnik M 2015 Absence of YbeY RNase compromises the growth and enhances the virulence plasmid gene expression of *Yersinia enterocolitica* O:3. Microbiology 161:285–299. doi:10.1099/mic.0.083097-0. 25416689

[11] Erental A, Kalderon Z, Saada A, Smith Y, Engelberg-Kulka H 2014 Apoptosis-like death, an extreme SOS response in *Escherichia coli*. mBio 5:e01426-14. doi:10.1128/mBio.01426-14. 25028428 PMC4161249

[12] Sykes MT, Williamson JR 2009 A complex assembly landscape for the 30S ribosomal subunit. Annu Rev Biophys 38:197–215. doi:10.1146/annurev.biophys.050708.133615. 19416066 PMC5654522

[13] Shajani Z, Sykes MT, Williamson JR 2011 Assembly of bacterial ribosomes. Annu Rev Biochem 80:501–526. doi:10.1146/annurev-biochem-062608-160432. 21529161

[14] Gupta N, Culver GM 2014 Multiple in vivo pathways for *Escherichia coli* small ribosomal subunit assembly occur on one pre-rRNA. Nat Struct Mol Biol 21:937–943. doi:10.1038/nsmb.2887. 25195050 PMC4355579

[15] Deutscher MP 2009 Maturation and degradation of ribosomal RNA in bacteria. Prog Mol Biol Transl Sci 85:369–391. doi:10.1016/S0079-6603(08)00809-X. 19215777

[16] Butland G, Peregrín-Alvarez JM, Li J, Yang W, Yang X, Canadien V, Starostine A, Richards D, Beattie B, Krogan N, Davey M, Parkinson J, Greenblatt J, Emili A 2005 Interaction network containing conserved and essential protein complexes in Escherichia coli. Nature 433:531–537. doi:10.1038/nature03239. 15690043

[17] Arifuzzaman M, Maeda M, Itoh A, Nishikata K, Takita C, Saito R, Ara T, Nakahigashi K, Huang H-C, Hirai A, Tsuzuki K, Nakamura S, Altaf-Ul-Amin M, Oshima T, Baba T, Yamamoto N, Kawamura T, Ioka-Nakamichi T, Kitagawa M, Tomita M, Kanaya S, Wada C, Mori H 2006 Large-scale identification of protein-protein interaction of Escherichia coli K-12. Genome Res 16:686–691. 16606699 10.1101/gr.4527806 PMC1457052

[18] Hu P, Janga SC, Babu M, Díaz-Mejía JJ, Butland G, Yang W, Pogoutse O, Guo X, Phanse S, Wong P, Chandran S, Christopoulos C, Nazarians-Armavil A, Nasseri NK, Musso G, Ali M, Nazemof N, Eroukova V, Golshani A, Paccanaro A, Greenblatt JF, Moreno-Hagelsieb G, Emili A 2009 Global functional atlas of *Escherichia coli* encompassing previously uncharacterized proteins. PLoS Biol 7:e96. doi:10.1371/journal.pbio.1000096. 19402753 PMC2672614

[19] Butland G, Babu M, Díaz-Mejía JJ, Bohdana F, Phanse S, Gold B, Yang W, Li J, Gagarinova AG, Pogoutse O, Mori H, Wanner BL, Lo H, Wasniewski J, Christopolous C, Ali M, Venn P, Safavi-Naini A, Sourour N, Caron S, Choi JY, Laigle L, Nazarians-Armavil A, Deshpande A, Joe S, Datsenko KA, Yamamoto N, Andrews BJ, Boone C, Ding H, Sheikh B, Moreno-Hagelseib G, Greenblatt JF, Emili A 2008 eSGA: *E. coli* synthetic genetic array analysis. Nat Methods 5:789–795. doi:10.1038/nmeth.1239. 18677321

[20] Babu M, Arnold R, Bundalovic-Torma C, Gagarinova A, Wong KS, Kumar A, Stewart G, Samanfar B, Aoki H, Wagih O, Vlasblom J, Phanse S, Lad K, Yeou Hsiung Yu A, Graham C, Jin K, Brown E, Golshani A, Kim P, Moreno-Hagelsieb G, Greenblatt J, Houry WA, Parkinson J, Emili A 2014 Quantitative genome-wide genetic interaction screens reveal global epistatic relationships of protein complexes in *Escherichia coli*. PLoS Genet 10:e1004120. doi:10.1371/journal.pgen.1004120. 24586182 PMC3930520

[21] Rajagopala SV, Sikorski P, Kumar A, Mosca R, Vlasblom J, Arnold R, Franca-Koh J, Pakala SB, Phanse S, Ceol A, Häuser R, Siszler G, Wuchty S, Emili A, Babu M, Aloy P, Pieper R, Uetz P 2014 The binary protein-protein interaction landscape of *Escherichia coli*. Nat Biotechnol 32:285–290. doi:10.1038/nbt.2831. 24561554 PMC4123855

[22] Battesti A, Bouveret E 2012 The bacterial two-hybrid system based on adenylate cyclase reconstitution in *Escherichia coli*. Methods 58:325–334. doi:10.1016/j.ymeth.2012.07.018. 22841567

[23] Karimova G, Pidoux J, Ullmann A, Ladant D 1998 A bacterial two-hybrid system based on a reconstituted signal transduction pathway. Proc Natl Acad Sci U S A 95:5752–5756. doi:10.1073/pnas.95.10.5752. 9576956 PMC20451

[24] Battesti A, Bouveret E 2006 Acyl carrier protein/SpoT interaction, the switch linking SpoT-dependent stress response to fatty acid metabolism. Mol Microbiol 62:1048–1063. doi:10.1111/j.1365-2958.2006.05442.x. 17078815

[25] Battesti A, Bouveret E 2009 Bacteria possessing two RelA/SpoT-like proteins have evolved a specific stringent response involving the acyl carrier protein-SpoT interaction. J Bacteriol 191:616–624. doi:10.1128/JB.01195-08. 18996989 PMC2620808

[26] Commichau FM, Rothe FM, Herzberg C, Wagner E, Hellwig D, Lehnik-Habrink M, Hammer E, Völker U, Stülke J 2009 Novel activities of glycolytic enzymes in *Bacillus subtilis*: interactions with essential proteins involved in mRNA processing. Mol Cell Proteomics 8:1350–1360. doi:10.1074/mcp.M800546-MCP200. 19193632 PMC2690492

[27] Roux CM, DeMuth JP, Dunman PM 2011 Characterization of components of the *Staphylococcus aureus* mRNA degradosome holoenzyme-like complex. J Bacteriol 193:5520–5526. doi:10.1128/JB.05485-11. 21764917 PMC3187390

[28] Zhang J, Inouye M 2002 MazG, a nucleoside triphosphate pyrophosphohydrolase, interacts with Era, an essential GTPase in *Escherichia coli*. J Bacteriol 184:5323–5329. doi:10.1128/JB.184.19.5323-5329.2002. 12218018 PMC135369

[29] Tu C, Zhou X, Tropea JE, Austin BP, Waugh DS, Court DL, Ji X 2009 Structure of ERA in complex with the 3′ end of 16S rRNA: implications for ribosome biogenesis. Proc Natl Acad Sci U S A 106:14843–14848. doi:10.1073/pnas.0904032106. 19706445 PMC2736428

[30] Sharma MR, Barat C, Wilson DN, Booth TM, Kawazoe M, Hori-Takemoto C, Shirouzu M, Yokoyama S, Fucini P, Agrawal RK 2005 Interaction of Era with the 30S ribosomal subunit implications for 30S subunit assembly. Mol Cell 18:319–329. doi:10.1016/j.molcel.2005.03.028. 15866174

[31] Zhan C, Fedorov EV, Shi W, Ramagopal UA, Thirumuruhan R, Manjasetty BA, Almo SC, Fiser A, Chance MR, Fedorov AA 2005 The YbeY protein from *Escherichia coli* is a metalloprotein. Acta Crystallogr Sect F Struct Biol Cryst Commun 61:959–963. doi:10.1107/S1744309105031131. PMC1978141 16511207

[32] Rasouly A, Ron EZ 2009 Interplay between the heat shock response and translation in *Escherichia coli*. Res Microbiol 160:288–296. doi:10.1016/j.resmic.2009.03.007. 19379808

[33] Liang W, Deutscher MP 2013 Ribosomes regulate the stability and action of RNase R. J Biol Chem 288:34791–34798. doi:10.1074/jbc.M113.519553. 24133211 PMC3843092

[34] Malecki M, Bárria C, Arraiano CM 2014 Characterization of the RNase R association with ribosomes. BMC Microbiol 14:34. doi:10.1186/1471-2180-14-34. 24517631 PMC3942186

[35] Domingues S, Moreira RN, Andrade JM, Dos Santos RF, Bárria C, Viegas SC, Arraiano CM 2015 The role of RNase R in trans-translation and ribosomal quality control. Biochimie 114:113–118. doi:10.1016/j.biochi.2014.12.012. 25542646

[36] Carpousis AJ 2007 The RNA degradosome of *Escherichia coli*: an mRNA-degrading machine assembled on RNase E. Annu Rev Microbiol 61:71–87. doi:10.1146/annurev.micro.61.080706.093440. 17447862

[37] Vogel J, Luisi BF 2011 Hfq and its constellation of RNA. Nat Rev Microbiol 9:578–589. doi:10.1038/nrmicro2615. 21760622 PMC4615618

[38] Mackie GA 2013 RNase E: at the interface of bacterial RNA processing and decay. Nat Rev Microbiol 11:45–57. doi:10.1038/nrmicro2930. 23241849

[39] Vesper O, Amitai S, Belitsky M, Byrgazov K, Kaberdina AC, Engelberg-Kulka H, Moll I 2011 Selective translation of leaderless mRNAs by specialized ribosomes generated by MazF in *Escherichia coli*. Cell 147:147–157. doi:10.1016/j.cell.2011.07.047:1-11. 21944167 PMC4894548

[40] Potrykus K, Cashel M 2008 (p)ppGpp: still magical? Annu Rev Microbiol 62:35–51. doi:10.1146/annurev.micro.62.081307.162903. 18454629

[41] Braeken K, Moris M, Daniels R, Vanderleyden J, Michiels J 2006 New horizons for (p)ppGpp in bacterial and plant physiology. Trends Microbiol 14:45–54. doi:10.1016/j.tim.2005.11.006. 16343907

[42] Inoue K, Alsina J, Chen J, Inouye M 2003 Suppression of defective ribosome assembly in a *rbfA* deletion mutant by overexpression of Era, an essential GTPase in *Escherichia coli*. Mol Microbiol 48:1005–1016. doi:10.1046/j.1365-2958.2003.03475.x. 12753192

[43] Sato S, Shimoda Y, Muraki A, Kohara M, Nakamura Y, Tabata S 2007 A large-scale protein protein interaction analysis in *Synechocystis* sp. PCC6803. DNA Res 14:207–216. doi:10.1093/dnares/dsm021. 18000013 PMC2779905

[44] Häuser R, Ceol A, Rajagopala SV, Mosca R, Siszler G, Wermke N, Sikorski P, Schwarz F, Schick M, Wuchty S, Aloy P, Uetz P 2014 A second-generation protein-protein interaction network of *Helicobacter pylori*. Mol Cell Proteomics 13:1318–1329. doi:10.1074/mcp.O113.033571. 24627523 PMC4014287

[45] Sulthana S, Deutscher MP 2013 Multiple exoribonucleases catalyze maturation of the 3′ terminus of 16S ribosomal RNA (rRNA). J Biol Chem 288:12574–12579. doi:10.1074/jbc.C113.459172. 23532845 PMC3642305

[46] Tu C, Zhou X, Tarasov SG, Tropea JE, Austin BP, Waugh DS, Court DL, Ji X 2011 The Era GTPase recognizes the GAUCACCUCC sequence and binds helix 45 near the 3′ end of 16S rRNA. Proc Natl Acad Sci U S A 108:10156–10161. doi:10.1073/pnas.1017679108. 21646538 PMC3121871

[47] Sato A, Kobayashi G, Hayashi H, Yoshida H, Wada A, Maeda M, Hiraga S, Takeyasu K, Wada C 2005 The GTP binding protein Obg homolog ObgE is involved in ribosome maturation. Genes Cells 10:393–408. doi:10.1111/j.1365-2443.2005.00851.x. 15836769

[48] Verstraeten N, Fauvart M, Versées W, Michiels J 2011 The universally conserved prokaryotic GTPases. Microbiol Mol Biol Rev 75:507–542. doi:10.1128/MMBR.00009-11. 21885683 PMC3165542

[49] Tomar SK, Dhimole N, Chatterjee M, Prakash B 2009 Distinct GDP/GTP bound states of the tandem G-domains of EngA regulate ribosome binding. Nucleic Acids Res 37:2359–2370. doi:10.1093/nar/gkp107. 19246542 PMC2673443

[50] Lamb HK, Thompson P, Elliott C, Charles IG, Richards J, Lockyer M, Watkins N, Nichols C, Stammers DK, Bagshaw CR, Cooper A, Hawkins AR 2007 Functional analysis of the GTPases EngA and YhbZ encoded by *Salmonella typhimurium*. Protein Sci 16:2391–2402. doi:10.1110/ps.072900907. 17905831 PMC2211706

[51] Chang D-E, Smalley DJ, Conway T 2002 Gene expression profiling of *Escherichia coli* growth transitions: an expanded stringent response model. Mol Microbiol 45:289–306. doi:10.1046/j.1365-2958.2002.03001.x. 12123445

[52] Lee JH, Lennon CW, Ross W, Gourse RL 2012 Role of the coiled-coil tip of *Escherichia coli* DksA in promoter control. J Mol Biol 416:503–517. doi:10.1016/j.jmb.2011.12.028. 22200485 PMC3288215

[53] Fan H, Hahm J, Diggs S, Perry JJ, Blaha G 2015 Structural and functional analysis of BipA, a regulator of virulence in enteropathogenic *Escherichia coli*. J Biol Chem 290:20856–20864. doi:10.1074/jbc.M115.659136. 26163516 PMC4543647

[54] deLivron MA, Robinson VL 2008 *Salmonella enterica* serovar Typhimurium BipA exhibits two distinct ribosome binding modes. J Bacteriol 190:5944–5952. doi:10.1128/JB.00763-08. 18621905 PMC2519513

[55] Sambrook J, Russell DW 2001 Molecular cloning: a laboratory manual. Cold Spring Harbor Laboratory, Cold Spring Harbor, NY.

[56] Casadaban MJ, Cohen SN 1979 Lactose genes fused to exogenous promoters in one step using a Mu-*lac* bacteriophage: *in vivo* probe for transcriptional control sequences. Proc Natl Acad Sci U S A 76:4530–4533. doi:10.1073/pnas.76.9.4530. 159458 PMC411611

[57] Wachi M, Umitsuki G, Shimizu M, Takada A, Nagai K 1999 *Escherichia coli cafA* gene encodes a novel RNase, designated as RNase G, involved in processing of the 5’ end of 16S rRNA. Biochem Biophys Res Commun 259:483–488. doi:10.1006/bbrc.1999.0806. 10362534

[58] Li Z, Deutscher MP 1995 The tRNA processing enzyme RNase T is essential for maturation of 5S RNA. Proc Natl Acad Sci U S A 92:6883–6886. doi:10.1073/pnas.92.15.6883. 7542780 PMC41434

[59] Li Z, Pandit S, Deutscher MP 1999 RNase G (CafA protein) and RNase E are both required for the 5′ maturation of 16S ribosomal RNA. EMBO J 18:2878–2885. doi:10.1093/emboj/18.10.2878. 10329633 PMC1171368

[60] Li Z, Pandit S, Deutscher MP 1999 Maturation of 23S ribosomal RNA requires the exoribonuclease RNase T. RNA 5:139–146. doi:10.1017/S1355838299981669. 9917073 PMC1369746

[61] Karimova G, Ullmann A, Ladant D 2000 A bacterial two-hybrid system that exploits a cAMP signaling cascade in *Escherichia coli*. Methods Enzymol 328:59–73. 11075338 10.1016/s0076-6879(00)28390-0

[62] Sali A, Blundell TL 1993 Comparative protein modelling by satisfaction of spatial restraints. J Mol Biol 234:779–815. doi:10.1006/jmbi.1993.1626. 8254673

[63] Comeau SR, Kozakov D, Brenke R, Shen Y, Beglov D, Vajda S 2007 ClusPro: performance in CAPRI rounds 6-11 and the new server. Proteins 69:781–785. doi:10.1002/prot.21795. 17876812

