# Supplementary figures and images for: Identification of YbeY-Protein Interactions Involved in 16S rRNA Maturation and Stress Regulation in Escherichia coli
Source: mBio. 2016 Nov 8;7(6):e01785-16. doi: 10.1128/mBio.01785-16 (PMC5101352; doi:10.1128/mBio.01785-16)

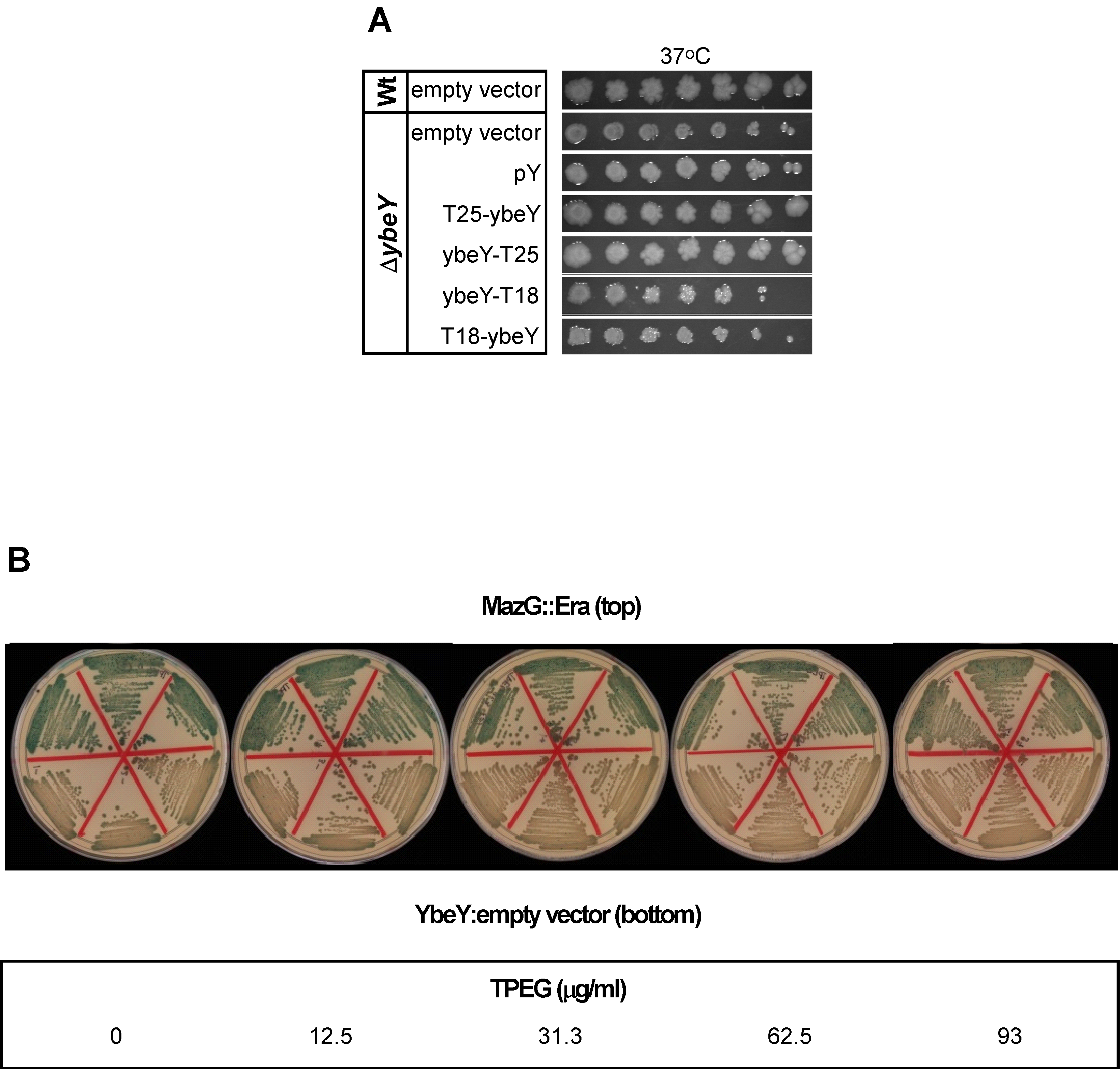

Supplement: Figure S1 — YbeY constructs used for B2H analysis. (A) Spotting assay of E. coli MC4100 ΔybeY transformed with the four B2H fusion constructs of YbeY used in this study (shown in Fig. 1B). Overexpression of fusion constructs does not substantially affect cell growth or viability. Only the high-copy-number plasmids expressing YbeY fused to the T18 fragment showed a slight reduction in growth. (B) Addition of TPEG to indicator plates reduced the formation of nonspecific background synthesis of β-galactosidase after prolonged incubation. Transformants of the E. coli B2H strain BTH101 (top, positive interaction; bottom, negative control) were streaked on plates containing 0, 12.5, 31.3, 62.5, or 93 µg/ml of TPEG and incubated at 30°C for 2 or 3 days. The highest TPEG concentration gave the cleanest background and was subsequently used in all experiments. Download [file mbo006163059sf1.tif]

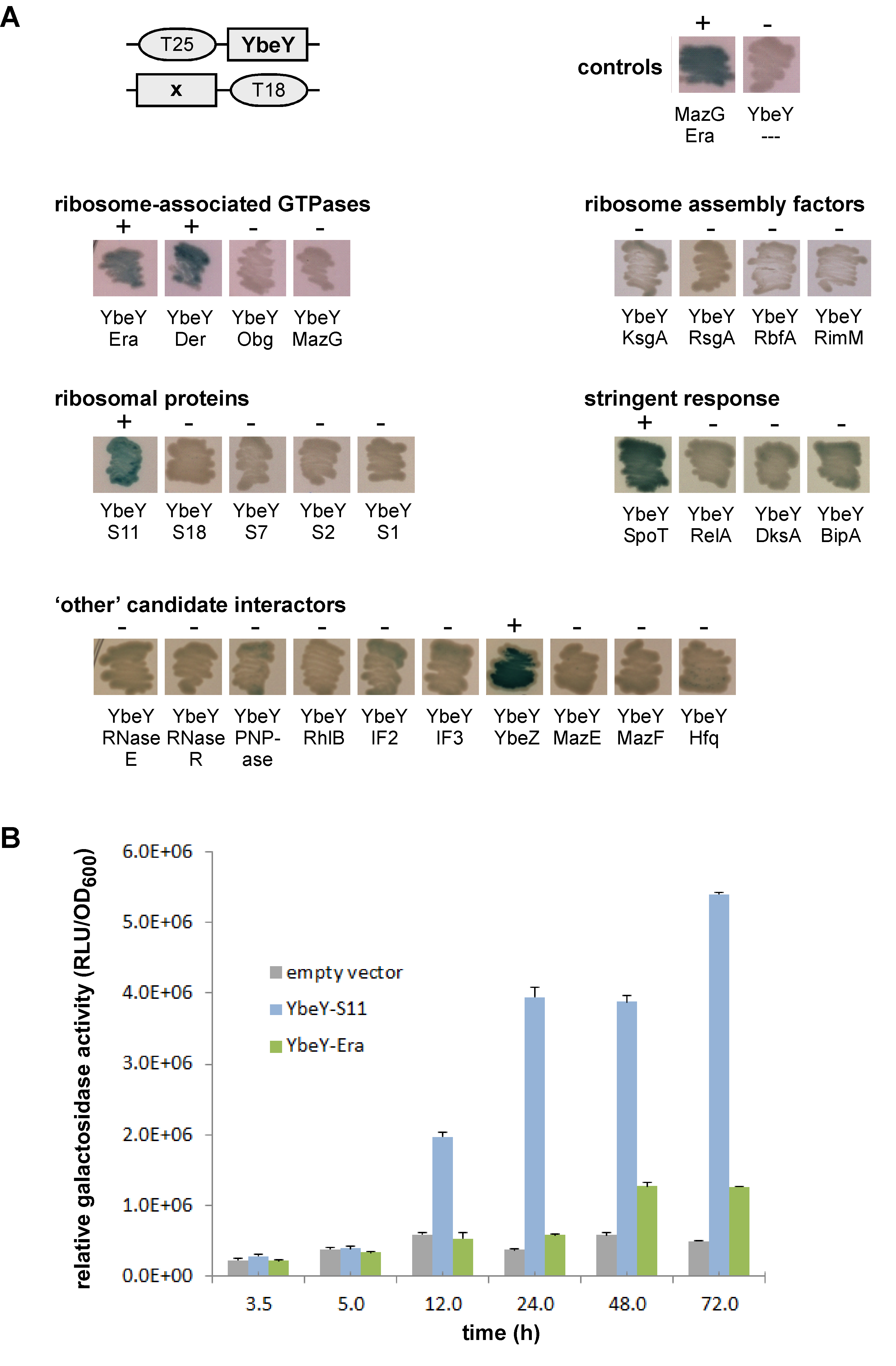

Supplement: Figure S2 — B2H analysis of interactions between YbeY and selected proteins of interest. (A) B2H analysis of YbeY (T25-YbeY) and selected candidate interaction partners (x-T18). YbeY shows interaction with the ribosome-associated GTPases Era and Der, ribosomal protein S11, the stringent response regulator SpoT, and YbeZ. Experiments were performed in triplicate, and representative streaks of BTH101 transformants are shown. The positive and negative controls were T25-MazG::Era-T18 and T25-YbeY::empty (T18), respectively. (B) Analysis of interaction between YbeY and S11 or Era in liquid culture using the T25-YbeY and x-T18 constructs. β-Galactosidase activity was measured over the course of 72 h and is given in relative luminescence units (RLU) normalized over cell density (OD600). Averages from six transformants are shown. Download [file mbo006163059sf2.tif]

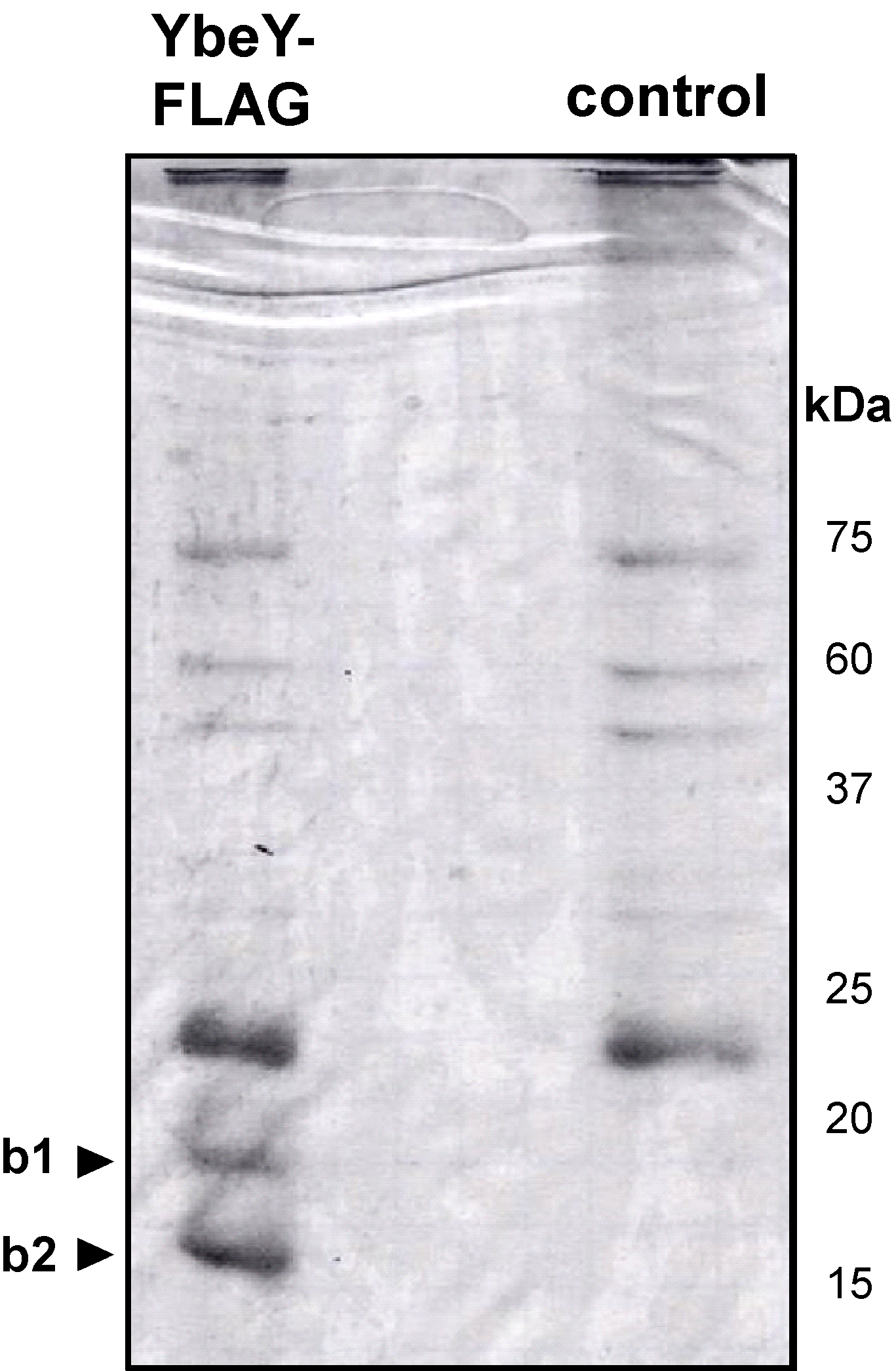

Supplement: Figure S3 — YbeY pulldown analysis. FLAG-tagged YbeY was immunoprecipitated from an E. coli MC4100 whole-cell lysate. Immunoprecipitates were separated by SDS-PAGE. Proteins in bands 1 and 2 were identified by MALDI-TOF mass spectrometry. Band 1 (b1) contained ribosomal proteins S7, S11, and L6. Band 2 (b2) contained subunit B of ATP synthase. An MC4100 strain carrying a non-FLAG-tagged YbeY was used as the control. The positions of size markers are shown in kilodaltons. Download [file mbo006163059sf3.tif]
